# Intrauterine Hematoma in the First Trimester and Pregnancy Complications: A Systematic Review and Meta-Analysis

**DOI:** 10.3389/fmed.2022.892146

**Published:** 2022-06-17

**Authors:** Zhao-juan Qin, Yu Xu, Yi Du, Ya-li Chen, Liang Sun, Ai Zheng

**Affiliations:** ^1^Department of Obstetrics and Gynecology, West China Second University Hospital, Sichuan University, Chengdu, China; ^2^Laboratory of Birth Defects and Related Diseases of Women and Children (Ministry of Education), Sichuan University, Chengdu, China

**Keywords:** intrauterine hematoma, singleton pregnancy, first trimester, prenatal complications, spontaneous abortion

## Abstract

**Background:**

Studies evaluating the relationship between intrauterine hematoma in the first trimester and prenatal complications are conflicting.

**Objectives:**

To evaluate whether intrauterine hematoma identified in the first trimester in women with singleton pregnancies is associated with adverse perinatal outcomes.

**Search Strategy:**

A comprehensive literature search of three databases (Embase, PubMed, and Web of Science) was performed up to September 2021.

**Selection Criteria:**

Cohort and case-control studies that have evaluated the relationship between intrauterine hematoma identified before 14 gestational weeks and the risk of prenatal complications, in women with a singleton pregnancy.

**Data Collection and Analysis:**

Two members of our team independently assessed the studies for inclusion, collected the data of interest, and assessed the risk of bias, and calculated pooled odds ratios (ORs) using random-effects models.

**Main Results:**

Nine studies, including 1,132 women with intrauterine hematoma and 11,179 controls met the inclusion criteria. Intrauterine hematoma increased the risk of spontaneous abortion [OR 2.15, 95% confidence interval (CI) 1.23–3.75], preterm birth (OR 1.83, 95% CI 1.37–2.43), fetal growth restriction (OR 2.33, 95% CI 1.13–4.83) and placental abruption (OR 3.16, 95% CI 1.23–8.13). No statistically significant association was found between intrauterine hematoma and preeclampsia (OR 1.30, 95% CI 0.87–1.94).

**Conclusion:**

Intrauterine hematoma in the first trimester of pregnancy increases the risk of spontaneous abortion, preterm birth, placental abruption, and fetal growth restriction.

**Systematic Review Registration:**

https://www.crd.york.ac.uk/PROSPERO/.

## Introduction

Intrauterine hematomas (IUHs) are commonly found on routine obstetric ultrasonography during the first trimester, however, they can be randomly observed throughout the pregnancy (1, 2). In ultrasound imaging, IUH often appears as a hypoechoic area between the uterine wall and chorionic membrane (2, 3). According to the literature, the incidence of IUH varies from 1 to 39.5% among previous studies, and this huge variation is mainly attributed to the heterogeneity in the study cohorts, definitions, ultrasonic equipment employed, and timing of diagnosis (4–8).

It was first proposed by Mantoni and Pedersen in 1981 (9), and several studies have sought to clarify the relationship between IUH and prenatal complications; however, the association remains uncertain and inconsistent. Some authors thought that compared with pregnant women who did not have IUH, women with IUH were not at a higher risk of adverse prenatal complications (10, 11). However, other researchers have contrasting opinions. They concluded that for women with IUH identified by ultrasonography, the risk of prenatal complications, including spontaneous abortion, premature labor, and fetal growth restriction (FGR) increases dramatically (12–14).

In 2011, Tuuli et al. (15) performed a systematic review and meta-analysis focus on the relationship between IUH and pregnancy complications. Seven studies, including 1,735 women with IUH and 70,703 controls, were included in their study. They reported that IUH significantly increased the risk of spontaneous abortion, stillbirth, placental abruption, preterm labor, and preterm premature rupture of membranes (PPROM) (15). They also found that IUH detected by ultrasound imaging during the first and second trimesters did not cause an increase in the incidence of FGR and preeclampsia (15). However, some limitations of this meta-analysis should be discussed. A major limitation of their study was the significant clinical heterogeneity among the included studies. Among these studies they included, the diagnosis time of IUH varied from 5 to 24 weeks of gestational age. In addition, the diagnostic criteria for IUH and definitions of the outcomes of interest were heterogeneous across studies. Following their systematic review, some high-quality studies on this topic have been published (4–6, 16–18).

Briefly, we believe that it is of clinical significance and necessary to conduct an updated literature review on this topic.

## Materials and Methods

This systematic review and meta-analysis were conducted following the recommendations of “The PRISMA 2020 statement: an updated guideline for reporting systematic reviews” (19) and “meta-analyses and systematic reviews of observational studies in epidemiology (MOOSE) group” (20). The protocol of this study was registered in PROSPERO (Registration no. CRD42020183315).

The literature search, eligibility identification, quality assessment, and data collection were performed independently by two or three members of our team. Any disagreements in these processes were resolved through discussion or, if necessary, consultation with a third senior researcher (Ai Zheng).

### Literature Search

Three electronic databases (Embase, PubMed, and Web of Science) were searched for eligible studies (database inception to 14 September 2021). Given our limited linguistic proficiency, the searches were limited to studies published in English journals. Studies that have reported the results of interest, regardless of the type, were included. Medical subject headings, keywords, and search strategies were tailored for each electronic database by Yu Xu, who specializes in medical literature retrieval. Gray literature resources, such as conference summaries identified by database searches and the reference lists of eligible studies, were reviewed and searched for potentially eligible studies. Step-by-step search strategies for Embase, PubMed, and Web of Science are presented in Supplementary Material 1.

### Study Selection

Retrieved records from the database and manual searches were managed using EndNote (version X9). The study selection process was independently conducted by two members (Zhaojuan Qin and Yu Xu) of our team. First, duplicate studies were excluded. Subsequently, an initial assessment of relevance was made by reviewing the titles and abstracts of the remaining records. Finally, the full text of the remaining records was reviewed for eligibility.

Observational studies (cohort studies, or case-control studies) were included if they met the following inclusion criteria: enrolled adult women (aged >18 years) with singleton intrauterine pregnancy; patients of the case-cohort had IUH identified by ultrasound imaging in the first trimester, and the women in the control cohort had normal pregnancies; pregnancy outcomes and complications were compared between the two cohorts; results were reported in the form of odds ratios (ORs) with 95% confidence intervals (CIs). The first trimester was defined as the time between the 1st day of the last menstrual period and the end of the 13th week of pregnancy. Gestational age was identified based on ultrasound biometric measurement (crown-rump length) of the fetus when the last menstrual period was unknown (21).

Studies were excluded for the following reasons: results were not reported in a peer-reviewed journal; results were reported in languages other than English; the study population was duplicated in another study included in final results analysis; the study cohort included women with multiple pregnancies; or the study cohort only included women whose pregnancy was achieved by assisted reproductive technology. When studies with duplicate cohorts were found, studies with more participating centers or larger sample sizes were included in our meta-analysis.

### Quality Assessment

The risk of bias of each eligible study was assessed using the guidelines of the “Newcastle-Ottawa Scale (NOS) for the assessment of the quality of non-randomised studies in meta-analysis” (22). The NOS estimates the risk of bias by assigning points to eight items that which are categorized into the following three domains: “selection of participants, measures of exposure and outcome variables, and appropriate control of confounding” (22). A star system was employed to enable a semi-quantitative assessment of the risk of bias (22). As is commonly accepted in previous studies (23–25), the risk of bias in a particular study is thought to be low if the NOS score is at least 7 points. Otherwise, the risk of bias was considered to be high. The quality assessment of this study was independently performed by Yi Du and Ya-li Chen, and any dispute was resolved by discussion.

### Data Extraction and Synthesis

A pre-designed spreadsheet was used for data extraction, and Zhao-juan Qin and Liang Sun independently collected the data collection step. The name of the first author, study design, size of the study cohort, year of publication, timing of IUH identification, IUH definition, number of events of interest, and ORs were extracted from all included studies. The collected data were reviewed and validated by a third investigator (Yu Xu). When data were unavailable in a publication, we made efforts to contact the corresponding author to obtain the missing details.

The pregnancy complications or outcomes of interest in this study were spontaneous abortion, preterm birth, FGR, placental abruption, and preeclampsia. Spontaneous abortion was defined as loss of pregnancy without external intervention before 28 weeks of gestation (26). Preterm birth was defined as delivery after 28 weeks of gestation but <37 weeks (27). FGR was defined as a birth weight less than the 10th percentile for gestational age, according to population norms. Placental abruption was defined as the removal of the placenta from the endometrium before delivery of the fetus (28). Preeclampsia was defined as blood pressure ≥140/90 mmHg intervals of more than 4 h apart, with proteinuria simultaneously, quantified by 24 h urine collection (> 3 g protein/24 h), after 20 weeks of gestation in a woman with previously normal blood pressure (29).

Separate meta-analyses were performed for each of the prenatal adverse outcomes, where possible. Heterogeneity was assessed statistically using the χ^2^ test and *I*^2^ value, and χ^2^ test for heterogeneity, and the extent of heterogeneity was quantified using the *I*^2^ value. An *I*^2^ statistic ≥50% and *P* < 0.1 indicated a high risk of heterogeneity. Random-effects models were used to combine ORs from different studies owing to the possibility of clinical heterogeneity. If there was significant heterogeneity among the included studies, possible sources of heterogeneity were investigated *via* sensitivity analysis. If the results of some original studies could not be statistically pooled by meta-analysis, they were presented in a tabular.

## Results

### Selection and Characteristics of Studies

**Figure 1:**
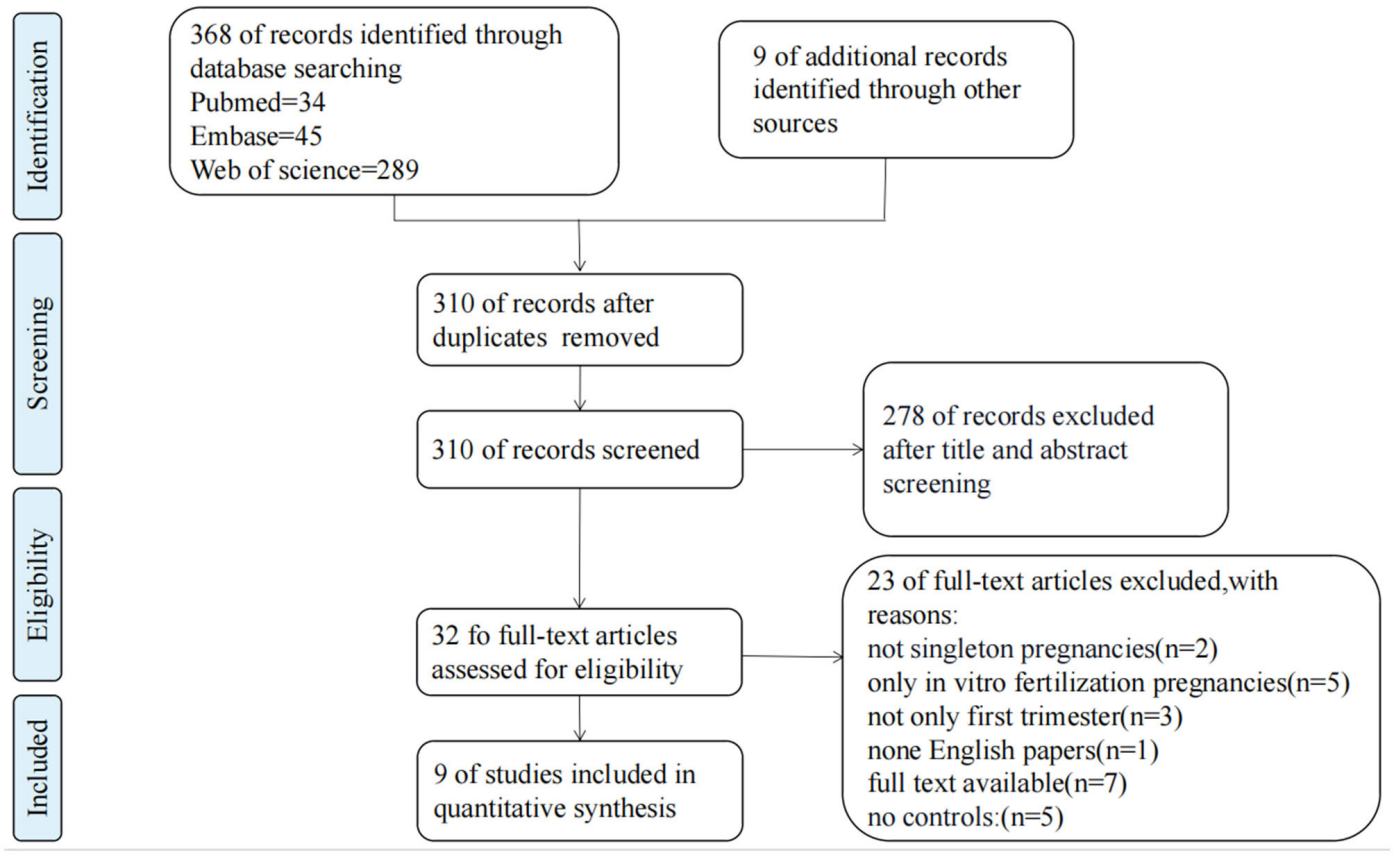
The flow chart of study selection.

Through literature searches, 377 records were identified in total. After duplicate studies were excluded, 310 literatures were reviewed the titles and abstracts, 32 literatures were screened in full text, and nine studies (4–6, 14, 16–18, 30, 31) with 12,311 patients involved were eventually included in data analysis. Characteristics of the 9 included articles are shown in Table 1. The study selection process is presented in Figure 1. These studies were published since 1996 to 2020, number of included samples ranged from 88 to 6,675. Among them, five studies were cohort studies and four were case-control studies.

**Table 1:** Characteristics of the studies included in the review.

**References**	**Study design**	**IUH**	**Control**	**Study participants**	**Exclusion criteria**
Al-Memar et al. (4)	Prospective cohort	268	678	Singleton intrauterine pregnancy; 5–14weeks	Women aged under 16 and over 50 years
Peixoto et al. (5)	Retrospective cohort	35	748	Singleton pregnancies with GA; the presence of IUH, 6–11weeks	Non-viable embryos without a detectable heartbeat and embryos with pathological features
Naert et al. (6)	Retrospective cohort	389	1,783	Singleton pregnancies; before 14 weeks	Women with pregnancy loss before 20 weeks of gestation. multiple gestations, a vanishing twin, or a fetal heart rate <100 beats per minute
Nagy et al. (14)	Prospective cohort	187	6,488	The presence of a viable, singleton gestation and delivery after 24 weeks' gestation	A non-viable fetus, multifetal pregnancy, or fetal abnormality diagnosed by ultrasonography
Palatnik et al. (16)	Retrospective cohort	512	1,024	Had a singleton non-anomalous gestation, before 14 weeks	Women with multifetal gestation, cerclage, or a uterine anomaly
EOzkaya et al. (17)	Case-contorl	43	45	Pregnancy between 7 and 14 weeks' gestation with vaginal bleeding	Patients with possible risk factors for primary end-points
Hashem et al. (18)	Case-contorl	100	200	Singleton viable intrauterine pregnancy, gestation, 6–14 weeks	Patients with a non-viable fetus, multifetal pregnancy, fetal abnormality, patients with history of recurrent miscarriage and with scarred uterus
Kurjak et al. (30)	Case-contorl	59	135	Vaginal bleeding, closed cervix, and ultrasonic findings of a living embryo and subchorionic hematoma	None
Johns et al. (31)	Case-contorl	51	78	Women with vaginal bleeding or lower abdominal pain at <12 weeks of gestation with SCH on ultrasound	multiple gestations, women with vaginal bleeding or referred for nuchal translucency measurement

### Assessment of Bias Risk

The overall quality of the studies included was acceptable, as shown in Table 2. Five of the studies had a medium risk of bias, and four had a low risk of bias. All cohort studies and case-control studies had a low risk of bias in terms of selection and comparability. All included studies had scores above 7 points, with a maximum score of 9 points.

**Table 2:** Risk of bias in the studies.

	**Study design**	**Selection**				**Comparability**	**Outcome**			
	**Cohort**	**Representative-ness of the exposed cohort**	**Non-exposed cohort**	**Ascertainment of exposure**	**Outcome of interest not present at start**	**Comparability of cohorts based on design or analysis**	**Assessment of outcome**	**Follow-up duration sufficient**	**Adequacy of follow-up**	**Risk of bias^a^**
Al-Memar et al. (4)	1	1	1	1	1	1	1	1	1	Low
Peixoto et al. (5)	1	1	1	1	1	1	1	1	0	Medium
Naert et al. (6)	1	1	1	1	1	1	0	1	1	Medium
Nagy et al. (14)	1	1	1	1	1	1	0	1	1	Medium
Palatnik et al. (16)	1	1	1	1	1	1	1	1	1	Low
	**Case-control**	**Adequate case definition**	**Representative-ness of cases**	**Selection of controls**	**Definition of controls**	**Definition of controls**	**Ascertainment of exposure**	**Same method of ascertainment for cases and controls**	**Non-response rate**	
Ozkaya et al. (17)	1	1	1	1	1	1	0	1	1	Medium
Hashem et al. (18)	1	1	1	1	1	1	1	1	1	Low
Kurjak et al. (30)	1	1	1	1	1	1	0	1	0	Medium
Johns et al. (31)	1	1	1	1	1	1	1	1	1	Low

a *Low, ≥7; medium, 5–7; high, ≤4*.

### Pooled Estimates of Outcomes of Interest

#### Spontaneous Abortion

**Figure 2:**
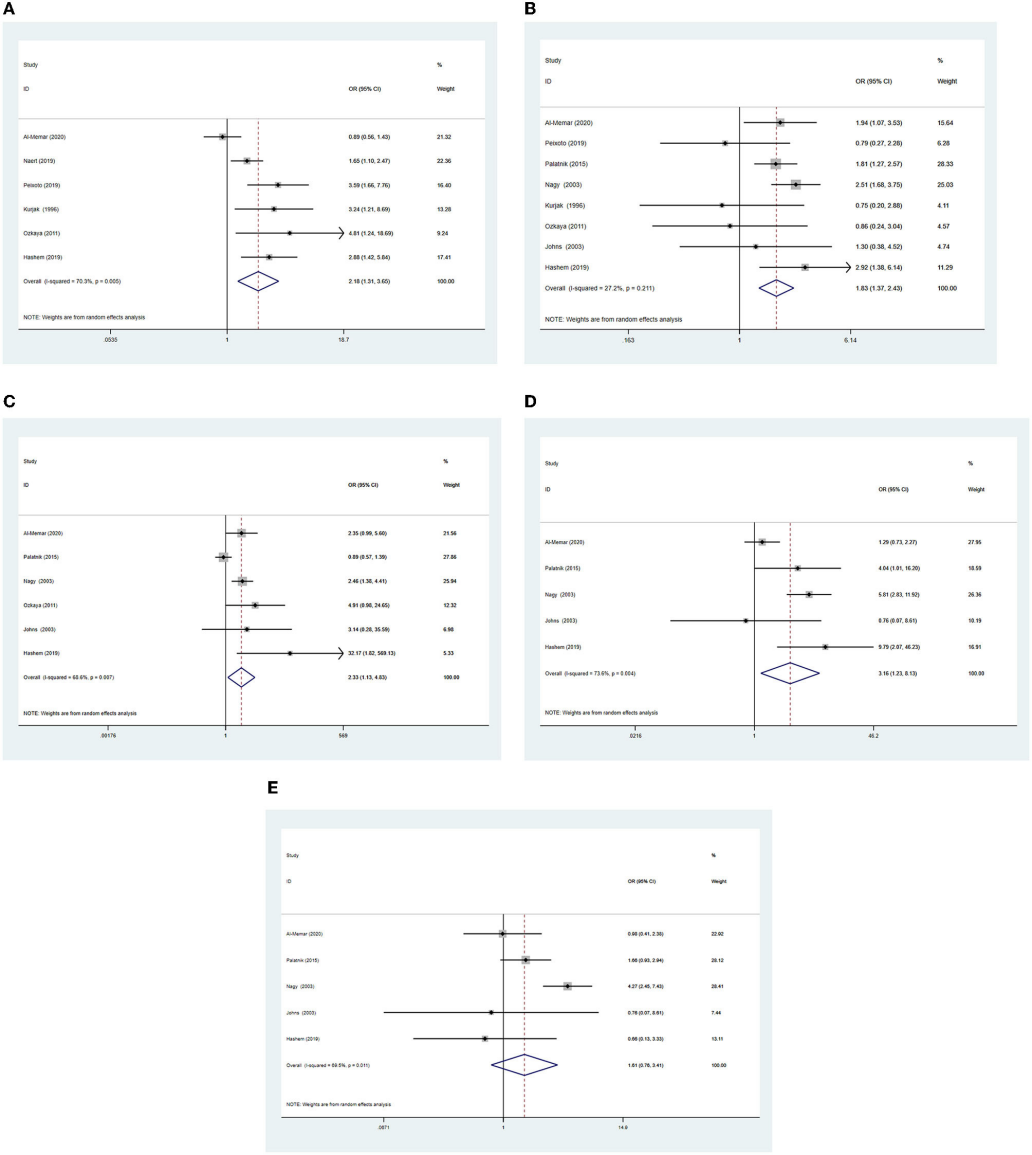
Forest plots of outcomes comparing the intrauterine hematoma group with the control group **(A)**, spontaneous abortion **(B)**, preterm birth **(C)**, fetal growth restriction **(D)**, placenta abruption **(E)**, pre-eclampsia.

Six studies (4–6, 17, 18, 30) reported the risk of spontaneous abortion in pregnant women diagnosed with IUH before 14 gestational weeks. The meta-analysis showed that for IUH in the first trimester of pregnancy, increases the likelihood of spontaneous abortion [odds ratio (OR) 2.17, 95% confidence interval (CI) 1.29–3.63] (Figure 2A). Significant heterogeneity was observed (χ^2^ = 20.90, *P* = 0.001, *I*^2^ = 75%). Accordingly, sensitivity analysis was performed to explore the influence of a single study on the pooled results of the meta-analysis. The results of the sensitivity analyses showed that the study by Al-Memar et al. (4) had a great influence on the results of the pooled synthesis. After excluding that study, the meta-analysis of the remaining five studies revealed that IUH was still associated with an increased risk of spontaneous abortion (OR 2.57, 95% CI 1.67–3.95), with low heterogeneity (*I*^2^ = 39%).

#### Preterm Birth

Eight studies (4, 5, 14, 16–18, 30, 31) reported the risk of preterm birth in pregnant women with IUH identified in the first trimester, and the pooled ORs using random-effects models revealed an increased risk of preterm birth (OR 1.83, 95% CI 1.37–2.43; Figure 2B) in women with IUH when compared to women with normal pregnancies. No significant heterogeneity was noted among these studies (χ^2^ = 9.62, *P* = 0.21, *I*^2^ = 27%).

#### Fetal Growth Restriction

Six studies (4, 14, 16–18, 31) reported FGR as an outcome. IUH was increased the likelihood of FGR (OR 2.33, 95% CI 1.13–4.83; Figure 2C). There was statistical heterogeneity among the studies (χ^2^ = 15.92, *P* = 0.007, *I*^2^ = 69%). Similarly, for the outcome of FGR, the pooled OR was unchanged when Palatnik's study (16) was excluded (OR 2.90, 95% CI 1.93–4.37), showing low heterogeneity (*I*^2^ = 0%).

#### Placental Abruption

Five studies (4, 14, 16, 18, 31) reported placental abruption as an outcome. IUH was also significantly increased the likelihood of placental abruption (OR 3.16, 95% CI 1.23–8.13; Figure 2D). Statistical heterogeneity was noted in placental abruption (χ^2^ = 15.12, *P* = 0.004, *I*^2^ = 74%). For the outcome of placental abruption, the pooled OR was unchanged when Al-Memar's study (4) was excluded (OR 5.13, 95% CI 2.71–9.71), demonstrating low heterogeneity *I*^2^ = 9%.

#### Preeclampsia

Five studies (4, 14, 16, 18, 31) reported preeclampsia as an outcome. However, no statistically association was observed between IUH and preeclampsia (OR 1.30, 95% CI 0.87–1.94; Figure 2E). There was statistical heterogeneity in preeclampsia (χ^2^ = 13.12, *P* = 0.01, *I*^2^ = 70%). Regarding the outcome of preeclampsia, the pooled OR was unchanged when Nagy's study (14) was excluded (OR 1.31, 95% CI 0.83–2.06), presenting low heterogeneity *I*^2^ = 0%.

## Discussion

Before the placenta structure has been formed, the exchange barrier between mother and fetus is the chorion. IUH is another common term for intrauterine bleeding. In IUH, ultrasound imaging detects a hematoma or hypoechoic hemorrhage between the uterine wall and the gestational sac, which is the separation of fetal membranes in the first trimester, also known as subchorionic hemorrhage. Most women present with mild vaginal bleeding, but some are asymptomatic on ultrasound imaging. In general, IUH is relatively common in clinical practice, with an incidence of 2.8% (14) to 28.3% (4) among the nine studies included in our meta-analysis. This may be due to the use of more advanced ultrasound equipment during the first trimester, which provides higher-quality images. The gestational age at the initial prenatal examination is earlier than in the past, and many asymptomatic IUH cases are detected early. In clinical practice, pregnant women with IUH are prone to anxiety. However, there is a lack of consensus on whether IUH found in the first trimester by ultrasonography increases the risk of prenatal adverse events in ongoing singleton pregnancies, for example, among studies assessing whether IUH increases the likelihood of spontaneous abortion in the first-trimester, some show an increased risk of spontaneous abortion (16, 18), whereas others elaborate no increased risk (4, 6, 31). For obstetricians, when dealing with counseling for pregnant women with IUH, the research conclusions of the current studies may be confusing. However, the present meta-analysis suggests that an IUH during the first trimester significantly increased the likelihood of spontaneous abortion in pregnant women, compared with normal pregnancy. In addition, IUH also increased the likelihood of preterm birth, placental abruption and FGR. The present study shows that IUH was not associated with the occurrence of preeclampsia.

According to current research results, there have been three systematic reviews on this topic (2, 15, 32). Pearlstone et al. (2) reported that a small IUH was common in the first trimester and did not increase the risk of pregnancy complications during pregnancy. While Tuuli et al. (15) concluded that IUH increased the risk of spontaneous abortion, stillbirth, preterm delivery, and PPROM. The latest one demonstrated that a retroplacental, posterior or subchorionic in the fundus of uterus, and/or persistent IUH is associated with adverse outcomes in the ongoing pregnancy (32). However, they included women in both the first trimester and second trimester of pregnancy. Xiang et al. (32) even inclusive the series case report, the case-control studies and cohort studies, and synthesis the results of different types of studies.

The strength of our study is that this is the first study to generalize the available evidence evaluating the relationship between prenatal complications of singleton pregnancies and IUH in the first trimester. In addition, previous systematic reviews identified and collected literature before January 2014. By contrast, the present review was based on an exhaustive search of high-quality research until September 2021. In comparison, our study had a larger number of participants, including pregnant women with IUH detected only on ultrasound examinations performed before 14 weeks of pregnancy. Therefore, we believe our results are more reliable than those obtained when comparing prenatal complications of singleton pregnancies with those of IUH in the first trimester.

Importantly, the conclusions of our study have significant health care practice implications, and we may provide information to obstetricians for future clinical practice decisions. Therefore, this meta-analysis can guide the clinical decision-making. Women with IUH before 14 weeks of gestation can be counseled about that they are at increased risk of spontaneous abortion. They should also be informed that they may develop placental abruption, FGR, an increased risk of preterm birth, and possibly receive more surveillance during pregnancy.

This study also has some limitations. A considered heterogeneity is estimated between studies, because of differences in diagnostic methods, criteria, and sampling frames. Regarding the influence of IUH on pregnancy outcomes, the diagnosis was made by transvaginal sonography or abdominal sonography. The location, volume, and duration of IUH, combined with threatened abortion symptoms such as vaginal bleeding and abdominal pain, may affect the pregnancy outcomes, and there may be mixed bias in related studies. Heller et al. (3) evaluated and compared several grading systems of IUH size in a study population of first-trimester pregnancies, and estimated IUH size in relation to the gestational sac size was superior to other methods of IUH quantification. Therefore, the size of the hematoma relative to the gestational sac size is considerably remarkable. There may be no complications in cases with small hematomas, but important complications may occur in those with larger hematomas. Hashem et al. (18) demonstrated that spontaneous abortion was more likely to occur with large hematomas than small-sized IUH, this was also supported by Ozkaya et al. (17). Due to the inconsistent grading standards of IUH size in our included literatures, we did not have enough data to discuss the impact of IUH size on prenatal complications. There may be differences in exposure and outcomes in terms of diagnostic methods and criteria. We only included literatures publications in English. Therefore, we minimized potential bias by having two independent reviewers screen eligible studies, extract data, and assess the quality of included studies.

At present, the research on the etiology of IUH is still unclear. Part of the reasons may be the external impact on the abdomen in the early pregnancy, or gestational hypertension during pregnancy, which may lead to the separation of part of the fetal membranes from the uterine wall. In addition, IUH may be more common in *in-vitro* fertilization pregnancies and multiple pregnancies (33). The length of the cervical canal also influences the separation of the fetal membranes. Taken together, our study suggests that women with IUH have an increased the likelihood of having adverse prenatal complications such as preterm birth, spontaneous abortion, placental abruption, and FGR, in the first trimester. In addition, further research is needed to investigate the possible mechanisms by which IUH is associated with antenatal adverse events.

## Conclusion

To the best of our knowledge, this is the first study to assess the association between prenatal complications in a single pregnancy and IUH in the first trimester of pregnancy. These results indicate that IUH in the first trimester of pregnancy increases the risk of preterm birth, spontaneous abortion, placental abruption, and FGR. Nonetheless, further studies are required to confirm this finding.

## Data Availability Statement

The original contributions presented in the study are included in the article/Supplementary Material, further inquiries can be directed to the corresponding author.

## Author Contributions

Z-jQ, YX, and AZ: conceptualization. YX and Z-jQ: methodology and writing—original draft. Z-jQ, YX, YD, Y-lC, and LS: data collection. YX and AZ: project administration and supervision. All authors contributed to manuscript revision, read, and approved the submitted version.

## Funding

This work was supported by the project of Scientific and Technological Department of Sichuan Province (Project No: 2019YFS0417).

## Conflict of Interest

The authors declare that the research was conducted in the absence of any commercial or financial relationships that could be construed as a potential conflict of interest.

## Publisher's Note

All claims expressed in this article are solely those of the authors and do not necessarily represent those of their affiliated organizations, or those of the publisher, the editors and the reviewers. Any product that may be evaluated in this article, or claim that may be made by its manufacturer, is not guaranteed or endorsed by the publisher.
