# Supplementary material for: Intrauterine Hematoma in the First Trimester and Pregnancy Complications: A Systematic Review and Meta-Analysis
Source: Front Med (Lausanne). 2022 Jun 17;9:892146. doi: 10.3389/fmed.2022.892146 (PMC9247277; doi:10.3389/fmed.2022.892146)
Supplement: Supplementary file 1 [file Data_Sheet_1.docx]

**S1. The details of search strategies.**

| Database | Search step | Search strategy | Search result |
| --- | --- | --- | --- |
| PubMed |  |  |  |
|  | #1 | "intrauterine hematoma"[Title/Abstract] OR "intrauterine hemorrhage"[Title/Abstract] OR "intrauterine bleeding"[Title/Abstract] OR (("intrauterin"[All Fields] OR "Intrauterine"[All Fields]) AND "collection"[Title/Abstract]) OR "intrauterine fluid"[Title/Abstract] OR "subchorionic hematoma"[Title/Abstract] OR "subchorionic hemorrhage"[Title/Abstract] OR "subchorionic bleeding"[Title/Abstract] OR ("subchorionic"[All Fields] AND "collection"[Title/Abstract]) OR "subchorionic fluid"[Title/Abstract] OR "subchorionic haematoma"[Title/Abstract] OR "retroplacental hematoma"[Title/Abstract] OR "retroplacental hemorrhage"[Title/Abstract] OR "retroplacental bleeding"[Title/Abstract] OR ("retroplacental"[All Fields] AND "collection"[Title/Abstract]) OR ("retroplacental"[All Fields] AND "fluid"[Title/Abstract]) OR "retroplacental haematoma"[Title/Abstract] OR ("subplacental"[All Fields] AND "hematoma"[Title/Abstract]) OR ("subplacental"[All Fields] AND "hemorrhage"[Title/Abstract]) OR ("subplacental"[All Fields] AND "bleeding"[Title/Abstract]) OR ("subplacental"[All Fields] AND "collection"[Title/Abstract]) OR ("subplacental"[All Fields] AND "fluid"[Title/Abstract]) OR ("subplacental"[All Fields] AND "haematoma"[Title/Abstract]) | 1281 |
|  |  |  |  |
|  | #2 | "adverse pregnancy outcome"[Title/Abstract] OR "pregnancy complication"[Title/Abstract] OR "pregnancy outcome"[Title/Abstract] OR "pregnancy induced hypertension"[Title/Abstract] OR "gestational hypertension"[Title/Abstract] OR "pre-eclampsia"[Title/Abstract] OR "placenta abruption"[Title/Abstract] OR "preterm birth"[Title/Abstract] OR "preterm delivery"[Title/Abstract] OR "small for gestational age"[Title/Abstract] OR "miscarriage"[Title/Abstract] OR "spontaneous abortion"[Title/Abstract] OR ("early"[Title/Abstract] AND "late pregnancy loss"[Title/Abstract]) OR "abruption"[Title/Abstract] OR "premature rupture of membranes"[Title/Abstract] OR "preterm premature rupture of membranes"[Title/Abstract] OR "fetal mortality"[Title/Abstract] OR "fetal distress"[Title/Abstract] OR "meconium stained amniotic fluid"[Title/Abstract] OR "low 5 min apgar score"[Title/Abstract] OR "nicu admission"[Title/Abstract] OR (("infant, newborn"[MeSH Terms] OR ("infant"[All Fields] AND "newborn"[All Fields]) OR "newborn infant"[All Fields] OR "newborn"[All Fields] OR "newborns"[All Fields] OR "newborn s"[All Fields]) AND "respiratory diseases"[Title/Abstract]) | 90426 |
|  |  |  |  |
|  | #3 | (first trimester[Title/Abstract]) OR (early pregnancy[Title/Abstract]) | 41386 |
|  |  |  |  |
|  | #4 | "prognosis"[MeSH Terms:noexp] OR "diagnosed"[Title/Abstract] OR "cohort*"[Title/Abstract] OR ("cohort effect"[MeSH Terms] OR ("cohort"[All Fields] AND "effect"[All Fields]) OR "cohort effect"[All Fields]) OR "cohort studies"[MeSH Terms:noexp] OR "predictor*"[Title/Abstract] OR "death"[Title/Abstract] OR "models statistical"[All Fields] | 2821250 |
|  |  |  |  |
|  | #5 | #1 and #2 and #3and #4 | 34 |
|  |  |  |  |
| Web of Science |  |  |  |
|  | #1 | (AB=(intrauterine haematoma)) OR TI=(intrauterine haematoma) OR (AB=(intrauterine hemorrhage)) OR TI=(intrauterine hemorrhage) OR (AB=(Intrauterine bleeding)) OR TI=(Intrauterine bleeding) OR (AB=(Intrauterine collection)) OR TI=(Intrauterine collection) OR (AB=(Intrauterine fluid)) OR TI=(Intrauterine fluid) OR (AB=(subchorionic hematoma)) OR TI=(subchorionic hematoma) OR (AB=(subchorionic hemorrhage)) OR TI=(subchorionic hemorrhage) OR (AB=(subchorionic bleeding)) OR TI=(subchorionic bleeding) OR (AB=(subchorionic collection)) OR TI=(subchorionic collection) OR (AB=(subchorionic fluid)) OR TI=(subchorionic fluid) OR (AB=(subchorionic haematoma)) OR TI=(subchorionic haematoma) OR (AB=(subamniotic hematoma)) OR TI=(subamniotic hematoma) OR (AB=(retroplacental hematoma)) OR TI=(retroplacental hematoma) OR (AB=(retroplacental hemorrhage)) OR TI=(retroplacental hemorrhage) OR (AB=(retroplacental bleeding)) OR TI=(retroplacental bleeding) OR (AB=(retroplacental collection)) OR TI=(retroplacental collection) OR (AB=(retroplacental fluid)) OR TI=(retroplacental fluid) OR (AB=(retroplacental haematoma)) OR TI=(retroplacental haematoma) OR (AB=(subplacental hematoma)) OR TI=(subplacental hematoma) OR (AB=(Subplacental hemorrhage)) OR TI=(Subplacental hemorrhage) OR (AB=(subplacental bleeding)) OR TI=(subplacental bleeding) OR (AB=(subplacental collection)) OR TI=(subplacental collection) OR (AB=(subplacental fluid)) OR TI=(subplacental fluid) OR (AB=(subplacental haematoma)) OR TI=(subplacental haematoma) | 5750 |
|  |  |  |  |
|  | #2 | (AB=(adverse pregnancy outcome)) OR TI=(adverse pregnancy outcome) OR (AB=(pregnancy complication)) OR TI=(pregnancy complication) OR (AB=(pregnancy outcome)) OR TI=(pregnancy outcome) OR (AB=(pregnancy-induced hypertension)) OR TI=(pregnancy-induced hypertension) OR (AB=(gestational hypertension)) OR TI=(gestational hypertension) OR (AB=(pre-eclampsia)) OR TI=(pre-eclampsia) OR (AB=(placenta abruption)) OR TI=(placenta abruption) OR (AB=(preterm birth)) OR TI=(preterm birth) OR (AB=(preterm delivery)) OR TI=(preterm delivery) OR (AB=(small for gestational age)) OR TI=(small for gestational age) OR (AB=(miscarriage)) OR TI=(miscarriage) OR (AB=(spontaneous abortion)) OR TI=(spontaneous abortion) OR (AB=(early and late pregnancy loss)) OR TI=(early and late pregnancy loss) OR (AB=(abruption)) OR TI=(abruption) OR (AB=(premature rupture of membranes)) OR TI=(premature rupture of membranes) OR (AB=(preterm premature rupture of membranes)) OR TI=(preterm premature rupture of membranes) OR (AB=(fetal mortality)) OR TI=(fetal mortality) OR (AB=(fetal distress)) OR TI=(fetal distress) OR (AB=(meconium-stained amniotic fluid)) OR TI=(meconium-stained amniotic fluid) OR (AB=(low 5-min Apgar score)) OR TI=(low 5-min Apgar score) OR (AB=(NICU admission)) OR TI=(NICU admission) OR (AB=(newborn respiratory diseases)) OR TI=(newborn respiratory diseases) | 193034 |
|  |  |  |  |
|  | #3 | (AB=(first trimester)) OR TI=(first trimester) OR (AB=(early pregnancy)) OR TI=(early pregnancy) | 83454 |
|  |  |  |  |
|  | #4 | (AB=(prognosis)) OR TI=(prognosis) OR (AB=(diagnosed)) OR TI=(diagnosed) OR (AB=(cohort)) OR TI=(cohort) OR (AB=(cohort effect)) OR TI=(cohort effect) OR (AB=(cohort studies)) OR TI=(cohort studies) OR (AB=(predictor)) OR TI=(predictor) OR (AB=(death)) OR TI=(death) OR (AB=("models, statistical")) OR TI=("models, statistical") | 2943051 |
|  |  |  |  |
|  | #5 | #1 and #2 and #3and #4 | 289 |
|  |  |  |  |
| Embase |  |  |  |
|  | #1 | intrauterine hematoma':ab,ti OR 'intrauterine haematoma':ab,ti OR 'intrauterine hemorrhage':ab,ti OR 'intrauterine bleeding':ab,ti OR 'intrauterine collection':ab,ti OR 'intrauterine fluid':ab,ti OR 'subchorionic hematoma':ab,ti OR 'subchorionic hemorrhage':ab,ti OR 'subchorionic bleeding':ab,ti OR 'subchorionic collection':ab,ti OR 'subchorionic fluid':ab,ti OR 'subchorionic haematoma':ab,ti OR 'subamniotic hematoma':ab,ti OR 'retroplacental hematoma':ab,ti OR 'retroplacental hemorrhage':ab,ti OR 'retroplacental bleeding':ab,ti OR 'retroplacental collection':ab,ti OR 'retroplacental fluid':ab,ti OR 'retroplacental haematoma':ab,ti OR 'subplacental hematoma':ab,ti OR 'subplacental hemorrhage':ab,ti OR 'subplacental bleeding':ab,ti OR 'subplacental collection':ab,ti OR 'subplacental fluid':ab,ti OR 'subplacental haematoma':ab,ti | 793 |
|  |  |  |  |
|  | #2 | 'adverse pregnancy outcome':ab,ti OR 'pregnancy complication':ab,ti OR 'pregnancy outcome':ab,ti OR 'pregnancy-induced hypertension':ab,ti OR 'gestational hypertension':ab,ti OR 'pre-eclampsia':ab,ti OR 'placenta abruption':ab,ti OR 'preterm birth':ab,ti OR 'preterm delivery':ab,ti OR 'small for gestational age':ab,ti OR 'miscarriage':ab,ti OR 'spontaneous abortion':ab,ti OR 'early and late pregnancy loss':ab,ti OR 'abruption':ab,ti OR 'premature rupture of membranes':ab,ti OR 'preterm premature rupture of membranes':ab,ti OR 'fetal mortality':ab,ti OR 'fetal distress':ab,ti OR 'meconium-stained amniotic fluid':ab,ti OR 'low 5-min apgar score':ab,ti OR 'nicu admission':ab,ti OR 'newborn respiratory diseases':ab,ti | 130,521 |
|  |  |  |  |
|  | #3 | 'prognosis':ab,ti OR 'diagnosed':ab,ti OR 'cohort':ab,ti OR 'cohort effect':ab,ti OR 'cohort studies':ab,ti OR 'predictor':ab,ti OR 'death':ab,ti | 3,688,224 |
|  |  |  |  |
|  | #4 | 'first trimester':ab,ti OR 'early pregnancy':ab,ti | 57854 |
|  |  |  |  |
|  | #5 | #1 and #2 and #3and #4 | 45 |
